# Non-muscle Myosin II: Role in Microbial Infection and Its Potential as a Therapeutic Target

**DOI:** 10.3389/fmicb.2019.00401

**Published:** 2019-03-04

**Authors:** Lei Tan, Xiaomin Yuan, Yisong Liu, Xiong Cai, Shiyin Guo, Aibing Wang

**Affiliations:** ^1^Hunan Provincial Key Laboratory of Protein Engineering in Animal Vaccines, Research and Development Center for Animal Reverse Vaccinology of Hunan Province, College of Veterinary Medicine, Hunan Agricultural University, Changsha, China; ^2^Institute of Innovation and Applied Research in Chinese Medicine, Hunan University of Chinese Medicine, Changsha, China; ^3^College of Food Science and Technology, Hunan Agricultural University, Changsha, China

**Keywords:** non-muscle myosin II, co-factors/receptors, mammalian cells, regulatory pathways, regulators, microbial-triggered discords

## Abstract

Currently, the major measures of preventing and controlling microbial infection are vaccinations and drugs. However, the appearance of drug resistance microbial mounts is main obstacle in current anti-microbial therapy. One of the most ubiquitous actin-binding proteins, non-muscle myosin II (NM II) plays a crucial role in a wide range of cellular physiological activities in mammals, including cell adhesion, migration, and division. Nowadays, growing evidence indicates that aberrant expression or activity of NM II can be detected in many diseases caused by microbes, including viruses and bacteria. Furthermore, an important role for NM II in the infection of some microbes is verified. Importantly, modulating the expression of NM II with small hairpin RNA (shRNA) or the activity of it by inhibitors can affect microbial-triggered phenotypes. Therefore, NM II holds the promise to be a potential target for inhibiting the infection of microbes and even treating microbial-triggered discords. In spite of these, a comprehensive view on the functions of NM II in microbial infection and the regulators which have an impact on the roles of NM II in this context, is still lacking. In this review, we summarize our current knowledge on the roles of NM II in microbial-triggered discords and provide broad insights into its regulators. In addition, the existing challenge of investigating the multiple roles of NM II in microbial infection and developing NM II inhibitors for treating these microbial-triggered discords, are also discussed.

## Introduction

Diseases caused by microbial infections including viruses and bacteria post a significant risk to public health, even resulting in social panic and huge economic loss duo to the outbreaks, such as Human immunodeficiency virus (HIV) and herpes simplex virus type 1 (HSV-1) (Looker et al., 2017; Bengtson et al., 2018). The most effective methods against microbial infections are vaccines and/or drugs. However, the emergence of drug-resistant strains and viral mutations in some cases are the main issues for antimicrobial means, as these antimicrobial ways mainly target microbial proteins or DNA (De Clercq, 2002). For this reason, novel therapeutic approaches, in particular, new targets should be further explored to develop antimicrobial drugs.

As intracellular parasitic pathogens, viruses and some bacteria utilize host co-factors including receptors to complete a series of life cycles. There are different receptors/co-factors on the host cell surface or inside the cell, which interact with the microbial proteins and participate in microbial entry, proliferation, and release. Therefore, these host proteins can be employed as potential antimicrobial drug targets. For instance, hepatitis B virus (HBV) utilizes heat shock protein 90 (HSP 90) to facilitate the formation of the HBV capsid through the interaction of HSP 90 and HBV core protein dimers, and treating HepG2.2.15 cells with HSP 90 inhibitors clearly decreases HBV replication by interfering with HBV capsid assembly and polymerase activity (Shim et al., 2011).

Non-muscle myosin II (NM II) is a molecular motor that provides force for cell movement via catalyzing hydrolysis of ATP and participates in a wide range of biological processes in many eukaryotic cells, such as cell adhesion, cell migration (Vicente-Manzanares et al., 2009), cell division (Ding and Woollard, 2017), and cell pinocytosis (Ding and Woollard, 2017). NM II forms a hexamer protein complex consisting of three pairs of polypeptides: two heavy chains (NMHC II, ∼200 kDa) that comprise two globular heads and an alpha-helical tail, a pair of regulatory light chains (∼20 kDa) that are involved in the regulation of NM II activity, and a pair of essential light chains (∼17 kDa) which stabilize the heavy chain conformation (Vicente-Manzanares et al., 2009; Zhang and Gunst, 2017). In mammals, the NM II family can be divided into three isoforms (NM IIA, NM IIB, and NM IIC) with their heavy chains encoded by MYH9, MYH10, and MYH14 genes, respectively (Conti and Adelstein, 2008). These three isoforms share high similarity at amino acid level and have distinct dynamic properties, thereby playing overlapping and different roles in the biological processes of eukaryotic cells (Newell-Litwa et al., 2015; Pecci et al., 2018). For instance, the function of NM IIA in visceral endoderm cell-cell adhesion was able to be replaced by NM IIB *in vivo*, while the isoform-specific role of NM IIA in mouse placenta formation could not be substituted (Wang et al., 2010), suggesting the existence of special function of NM II isoforms in certain tissues/cells (Ma et al., 2010; Wang et al., 2010). As an actin-binding protein, NM II is ubiquitously expressed in various mammalian cell types and tissues (Newell-Litwa et al., 2015). However, the contents and distribution of those three isoforms of NM II are different in mammalian tissues/cells (Pecci et al., 2018). Relative high abundance of NM IIA (∼100%) but not NM IIB or NM IIC is detected in mouse spleen, while the relative abundance of NM IIB (∼65%) is higher than those of NM IIA (∼29%) or NM IIC (∼6%) in mouse spinal cord (Golomb et al., 2004). Additionally, the relative abundance of NM IIA is higher in Human Hela and HT29 cell types, but lower in Cos-7 cell type, compared with other two isoforms (Pecci et al., 2018). Notably, aberrant expression/activity of NM II has been detected in many microbial-triggered discords including virus-triggered discords and bacteria-triggered discords. For example, NM IIA is found to interact with Gn proteins of severe fever with thrombocytopenia syndrome virus (SFTSV) and its total expression is also augmented during SFTSV infection, while the application of siRNA specially inhibiting NM IIA expression suppresses the viral infection of cells, suggesting that SFTSV may utilize NM IIA to promote the efficiency of viral infection (Sun et al., 2014).

Considering the involvement of NM II in many physiological processes, a tight association of it with a range of disease pathologies has been established (Roberts and Baines, 2011). In particular, the participation of NM II in microbial-triggered discords has become a new focus in this field, as new findings have been obtained and important progress has recently been made. Additionally, the exploration of NM II as a therapeutic target has achieved fruitful results and novel regulators including inhibitors involved in the regulation of NM II’s expression and activity have been identified. Therefore, in this review we intend to provide an updated summary of such new information, with an emphasis on the involvement of NM II and NM II-related pathways in microbial-triggered discords, as well as its novel regulators.

## NM II in Microbial-Triggered Discords

**FIGURE 1:**
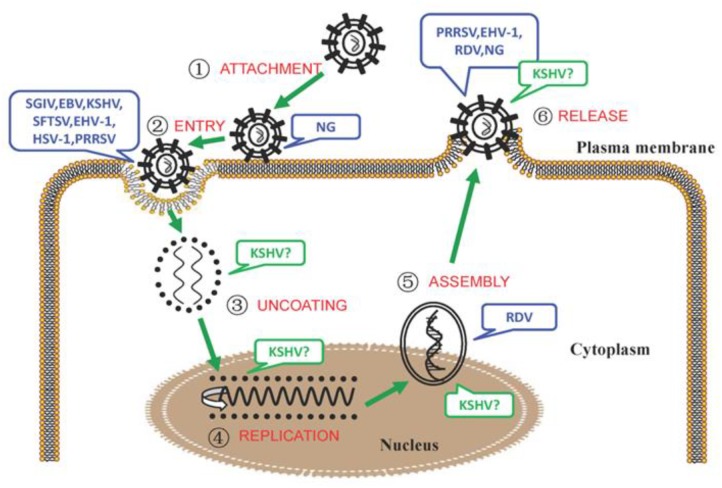
The involvement of NM II in the different processes of microbial infection.
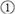
During the process of intracellular bacterial attachment, the potential roles of NM IIA in NG (*Neisseria gonorrhoeae*) adhesion to the target cells (Wang et al., 2017) were determined;
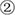
In the process of viral entry, the interactions between the glycoprotein H/glycoprotein L (gH/gL) protein of EBV and the C-terminal 1,665–1,960 aa region of NM IIA (Xiong et al., 2015); the adaptor protein (c-Cbl) and NM IIA (KSHV) (Valiya Veettil et al., 2010); the HSV-1 glycoprotein B and NM IIA, NM IIB (Arii et al., 2010, 2015); the glycoprotein Gn of SFTSV and NM IIA (Sun et al., 2014); and the glycoprotein GP5 of PRRSV and the C-terminal domain of NM IIA (Gao et al., 2016) were confirmed. In addition, the potential roles of NM II in EHV-1 (Cymerys et al., 2016), SGIV (Wang et al., 2014) entry were also speculated;
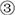
During the process of KSHV particle uncoating (Valiya Veettil et al., 2010), the vital roles of NM IIA were conjectured;
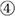
During the process of viral replication, the potential roles of NM IIA in KSHV infection (Zhu et al., 2005; Lyman and Enquist, 2009) were surmised;
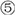
During the process of viral intracellular capsid assembling and transport, the vital roles of NM IIA in KSHV (Valiya Veettil et al., 2010), RDV (Wei et al., 2008b) infection were suggested;
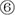
During the process of microbial release, the essential roles of NM IIA/NM II in KSHV (Valiya Veettil et al., 2010), EHV-1 (Cymerys et al., 2016), PRRSV (Guo J. et al., 2016), RDV (Wei et al., 2008b), and NG (Wang et al., 2017) intercellular spread were concluded. Note: The infection of microorganisms that NM II or its specific isoform was involved was marked with blue, that NM II or its specific isoform might be involved was marked with green and a “?”.

The microbial-triggered discords in which NM II has been identified to participate are summarized in Table 1, also shown in Figure 1 and separately illustrated in the following.

**Table 1:** Involvement of non-muscle myosin II (NM II) in microbial-triggered discords.

Pathogen	Alteration of NM II expression or activity	Reference
EBV	Increased NM IIA expression in nasopharyngeal epithelial cells	Xiong et al., 2015
KSHV	Increased activity and expression of NM IIA in HMVEC-d cells; Induced phosphorylation of NM IIA	Valiya Veettil et al., 2010; Bandyopadhyay et al., 2014
HSV-1	Increased both NM IIA and NM IIB cell-surface expression on Vero cells; Induced redistribution of NM IIA from the cytoplasm to the cell surface	Arii et al., 2010, 2015
EHV-1	Increased NMII activity in murine neurons	Cymerys et al., 2016
GIV	Induced variable expression of NM IIA	Yeh et al., 2008
HIV-1	Decreased NM IIA expression in podocyte, murine and human glomerular	Hays et al., 2012
SFTSV	Increased NM IIA expression; Induced redistribution of NM IIA from the cytoplasm to the cell surface	Sun et al., 2014
PRRSV	Increased NM IIA expression; Induced redistribution of NM IIA from the cytoplasm to the cell surface	Gao et al., 2016
RDV	Increased NM II expression	Wei et al., 2008b
LM	Increased activity and expression of NM IIA; Induced tyrosine-phosphorylation of NM IIA	Almeida et al., 2015
NG	Increased NM II activity at pathogen adherent site	Wang et al., 2017


*EBV, Epstein Barr virus; KSHV, Kaposi’s sarcoma-associated herpesvirus; HSV-1, Herpes simplex virus type 1; EHV-1, Equid herpesvirus type 1; GIV, Grouper iridovirus; HIV-1, Human immunodeficiency virus type 1; SFTSV, Severe fever with thrombocytopenia syndrome virus; PRRSV, Porcine reproductive and respiratory syndrome virus; RDV, Rice dwarf virus; LM, Listeria monocytogenes; NG, Neisseria gonorrhoeae*.

### NM II in Virus-Triggered Discords

#### Herpesvirus

The *Herpesvirus* family, which can affect a variety of organisms including humans, fish, frogs and reptiles, contains over 150 enveloped viruses with double-stranded linear DNA encoding 80 to 100 open reading frames (ORFs) (Boss et al., 2009; Roberts and Baines, 2011). According to the host tissue specificity and replication features, this family can be divided into three subfamilies, namely, α-, β-, and γ-herpesvirina (Roberts and Baines, 2011). Notably, Herpes simplex virus type 1 (HSV-1) and Equid herpesvirus type 1 (EHV-1) of α-herpesvirina, Epstein-Barr virus (EBV) and Kaposi’s sarcoma-associated herpesvirus (KSHV) of γ-herpesvirina, have been extensively studied due to their leading infection to humans or animals. The earliest report that NM II is involved in the infection of the *Herpesvirus* family was described by van Leeuwen et al. (2002). Thereafter, accumulating evidence indicates that NM II is implicated in the infection of this virus family and the role/function of NM II during virus infection may be distinct depending on the virus types and infectious processes as illustrated in the following sections.

#### Epstein-Barr Virus (EBV)

Epstein-Barr virus (EBV) is a nearly ubiquitous pathogen that causes damage to the health of human beings as EBV infects almost 90% of the global population (Hau and Tsao, 2017). EBV infection is associated with approximately 1.5% of all cancers including Hodgkin’s lymphoma and Burkitt’s lymphoma (Hau and Tsao, 2017; Teow et al., 2017). The implication of NM II in EBV infection was revealed by a recent report. In a series of exploratory experiments, Xiong et al. (2015) first discovered that immunoprecipitation with myc-tagged EBV gH/gL can pull down a 250 kDa protein in EBV infected sphere-like cell (SLCs) lysates and this protein is identified to be non-muscle myosin heavy chain IIA (NMHC IIA), suggesting that EBV gH/gL may interact with NM IIA, as further confirmed in subsequent co-immunoprecipitation and GST pull-down assays (Xiong et al., 2015). Moreover, the interaction of EBV gH/gL with NM IIA is verified to be mediated by the C-terminal 1,665–1,960 amino acids region of NM IIA. Co-localization assay also reveals that EBV infection leads to the redistribution NM IIA to the cell membrane and allows it to more extensively colocalized with the proteins of EBV (Xiong et al., 2015). Additionally, down-regulation of endogenous NM IIA expression by a NM IIA siRNA-mediated knock-down assay and a blocking assay with NMHC IIA antibody lowers the entry efficiency of EBV virions in nasopharyngeal epithelial cells, while over-expression of NM IIA in the cell membrane but not cytoplasm can significantly promote EBV infection efficiency (Xiong et al., 2015). These findings not only demonstrate the important role of NM IIA in mediating the entry of EBV into its target cells, but also imply that any inhibitor interrupting the interaction of NM IIA with the proteins gH/gL, gB, or BMF2 of EBV holds the promise to be an effective agent for preventing EBV entry or infection.

#### Kaposi’s Sarcoma-Associated Herpesvirus (KSHV)

Kaposi’s sarcoma-associated herpesvirus (KSHV), etiologically relevant to various tumors including Kaposi’s sarcoma (KS), plasmablastic lymphoma, and primary effusion lymphoma (PEL), can infect various target cells both *in vivo* and *in vitro* via diverse patterns of endocytosis. Among these ways, macropinocytosis is regarded as a major route of entry for KSHV and many other viruses (Valiya Veettil et al., 2010). During KSHV entry, the multi-domain adaptor protein of c-Cbl is found to play a major role in membrane blebbing and macropinocytosis (Valiya Veettil et al., 2010). Moreover, immunoprecipitation of c-Cbl with the lysates from KSHV-infected cells followed by mass spectrometry identifies NM IIA as a molecular partner of c-Cbl. The direct interaction of c-Cbl with NM IIA is mediated by the C-terminal region encompassing the proline rich domain (PRD) of c-Cbl. Furthermore, this interaction between c-Cbl and NM IIA is critical for triggering bleb-associated macropinocytosis of KSHV. Either inhibiting NM IIA ATPase activity by its inhibitor blebbistatin or silencing c-Cbl with shRNA, leads to the decreased entry and infection efficiency of KSHV virions (Valiya Veettil et al., 2010). Notably, NM IIA mediated bleb formation in KSHV macropinocytosis is also regulated by other cellular factors, e.g., tyrosine kinase EphrinA2 (EphA2) (Chakraborty et al., 2012), and calcium and integrin binding protein-1 (CIB1). For instance, CIB1, widely expressed in human tissues, is crucial for KSHV entry by promoting the activity of EphA2 to facilitate the interaction of NM IIA with the cytoskeletal cross linker alpha actinin 4, thereby providing the mechanical forces for macropinocytosis (Bandyopadhyay et al., 2014). Additionally, NM IIA is found inside the KSHV virions, suggesting that it may participate in intracellular capsid assemble, transportation and viral egress of KSHV (Zhu et al., 2005; Lyman and Enquist, 2009). However, further investigations are required to substantiate the roles of NM II in these processes.

#### Herpes Simplex Virus Type 1 (HSV-1)

Herpes simplex virus type 1 (HSV-1) is a world-spread pathogen which infects over a half of adult humans, resulting in diverse ocular, oral, and genital manifestations (Antoine and Shukla, 2014; Lathe and Haas, 2016). Currently, we still have no effective means for treating HSV-1. HSV-1 infection is regarded as a crucial factor for increasing the possibility of being infected with human immunodeficiency virus (HIV), which is characterized by lymphadenectasis and fever with high mortality (Han et al., 2018). An earlier study suggested that NM II may play a role in virus transport and egress during the virus life cycle of HSV-1. This speculation is mainly based on the following observations. NM IIA is firstly found to interact with the HSV-1 major tegument protein VP22 (van Leeuwen et al., 2002). Furthermore, HSV-1 infection leads to the reorganization of NM IIA. Meanwhile, blocking NM II ATPase activity using a myosin-specific inhibitor dramatically reduces the yield of extracellular HSV-1 virus production (van Leeuwen et al., 2002). Subsequent investigations further indicated that members of the NM II family were also involved in other aspects of HSV-1 infection and had overlapping functions during these processes (Arii et al., 2010, 2015). In the course of HSV-1 entering into host cells, both NM IIA and NM IIB are employed as cellular receptors/factors by directly associating with the HSV-1 envelope glycoprotein B (gB) on the cellular surface (Arii et al., 2010, 2015). The HL 60 cell line is relatively insensitive to HSV-1 infection (Pientong et al., 1989). However, over-expression of NM IIA improves the susceptibility of HL60 cells to HSV-1 infection (Arii et al., 2010). On the contrary, antibody blockage and down-regulation of NM IIA in permissive cells inhibit HSV-1 infection. In accordance with the role of NM IIA, knockdown of NM IIB expression in cultured cells that are sensitive to HSV-1 infection significantly suppresses the susceptibility to HSV-1 infection at viral entry and cell-to-cell fusion. On the other hand, up-regulation of NM IIB in target cells dramatically increases their susceptibility to HSV-1 infectivity (Arii et al., 2015). Though more fundamental studies are required, the ubiquitous expression of NM IIA and IIB, together with these important findings above, make them a target for developing medicinally relevant drugs to prevent HSV-1 infection.

#### Equid Herpesvirus Type 1 (EHV-1)

Equid herpesvirus type 1 (EHV-1) is the main pathogen that affects horses worldwide, causing huge economic loss in the horse industry (Lunn et al., 2009). EHV-1 virions are mainly transmitted from the lymphoid tissue of the upper respiratory tract to the central nervous system, the uterus as well as monocytes which regulate viremia (Shakya et al., 2017), thereby resulting in various clinical symptoms including respiratory disease, abortion, and neonatal death (Lunn et al., 2009; Dunowska, 2014). During EHV-1 infection in cultured cells, the actin cytoskeleton of the infected cells is induced to rearrange its distribution and intimately contact neighboring cells to promote the spread of virions from cell to cell (Dunowska, 2014). A recent study indicated that both application of blebbistatin or 2, 3-butanedione monoxime (BDM) which are well-recognized NM II inhibitors before and after EHV-1 infection are negative factors for viral entry and egress (Cymerys et al., 2016), respectively. Therefore, it is suggested that EHV-1 utilizes NM II and NM II-associated proteins for viral entry and the egress of progeny virions (Cymerys et al., 2016). However, further studies should be implemented for substantiating what kind of NM II isoforms is essential for EHV-1 infection.

#### Iridoviridae

The *Iridoviridae* family can be divided into five genera: *Ranavirus, Lymphocystivirus*, *Megalocytivirus*, *Iridovirus*, and *Chloriridovirus* (Murphy et al., 2012). Members of *Iridoviridae*, having a single double-stranded DNA with icosahedral cytoplasm, infect a wide variety of invertebrates and poikilothermic vertebrates, e.g., fish, insects, reptiles, and amphibians. Outbreaks of *Iridoviridae* infection in aquaculture have been reported in recent years and cause severe economic losses to cultured fish worldwide (Jeong et al., 2008; Dong et al., 2017).

Infectious spleen and kidney necrosis virus (ISKNV) of *Iridovirus* genera, Grouper iridovirus (GIV) and Singapore grouper iridovirus (SGIV) of *Ranavirus* genera that mainly infect mandarin and grouper fish, respectively, have initially been studied. The mechanisms of action between the host cells and *Iridoviridae* are intricate. Xu et al. (2011) showed that VP15R protein encoded by the fifteenth ORF of ISKNV is firstly transcribed within 12 h post infection and is verified to be able to bind to the heavy chains of NM II from zebra fish, mice and humans during ISKNV infection. Furthermore, Wang et al. (2014) demonstrated that blocking NM II activity using a NM II kinase inhibitor (ML-7) has a negative impact on SGIV entering into a host cell. Additionally, the transcriptional expression level of NM II A is changeable in GIV-infected grouper kidney (GK-2) cells at different time quantum (Yeh et al., 2008). Though these initial observations suggest a role for NM II in the viral infection of *Iridoviridae*, the exact role/function of NM II requires further investigation too.

#### Other Viruses

##### Human immunodeficiency virus type 1 (HIV-1)

Human immunodeficiency virus type 1 (HIV-1) is an enveloped and single-stranded positive sense RNA virus of the *Retrovirus* family, within the order *Lentivirus*. The genome of HIV-1 is approximately 9 kb in length, only encoding a transcription unit and expressing 15 proteins (Stoltzfus, 2009; Hidalgo and Swanson, 2017). HIV-1 infection is a major threat to humans globally, with especially high morbidity and mortality in sub-Saharan Africa. People of African descent infected with HIV-1 is prone to HIV-associated nephropathy (HIVAN) (Husain et al., 2018; Rednor and Ross, 2018). Accumulating evidence indicates that the genetic variants or aberrant expression of MYH9 gene which encodes the heavy chains of NM IIA is closely related with HIVAN (Hays et al., 2012; Colares et al., 2014).

MYH9 (the gene encoding the heavy chains of NM IIA) is found to be abundantly expressed in glomeruli, and specifically podocytes of human tissue. However, glomerular expression of MYH9 was reduced in the kidneys of the transgenic mice that ubiquitously express the HIV provirus genome that lacks gag/pol genes. Furthermore, MYH9 expression was also decreased in the podocytes from these transgenic mice aforementioned or the podocytes transduced with pseudotyped lentivirus containing pNL4-3 Δgag/pol (HIV-1)-EGFP vectors (Hays et al., 2012). Similarly declined expression of MYH9 was also observed in human podocytes transduced with HIV-1. Additionally, the expression of MYH9 was markedly reduced in human glomeruli in the setting of HIVAN (Hays et al., 2012). By using community structure analysis, further evidence showed that NM IIA interacts with protein networks including those of Rho, which mediates podocyte cytoskeletal structure and function, and networks regulated by the HIV-1 gene *nef* (a key mediator of podocytopathy in HIVAN), as well as pathways less well characterized in podocytes. Notably, HIV *nef* regulates signaling cascade including Rho proteins, while Rho pathway stabilizes the formation of actin-myosin filaments. Moreover, the involvement of NM IIA in the Rho cytoskeletal regulating pathway is confirmed (Hays et al., 2012, 2014). Therefore, it is speculated that the reduction of NM IIA expression may be of significance to HIV-1 infection and the pathogenesis of HIVAN (Hays et al., 2012, 2014). Though these observations imply that NM II can be the disturbance target for preventing or treating the infection of HIV, these initial findings warrant further investigation.

##### Severe fever with thrombocytopenia syndrome virus (SFTSV)

Severe fever with thrombocytopenia syndrome virus (SFTSV) is a novel enveloped, single-stranded negative sense RNA virus of the *Bunyaviridae* family. A case of SFTSV infection was first reported in hilly areas of Henan province of central China in 2009 (Yu et al., 2011). Subsequently, several deaths due to SFTSV infection were confirmed in Japan and South Korea (Kim et al., 2013; Takahashi et al., 2014). Disease manifestations linked to SFTSV include weakness, hemorrhagic fever, thrombocytopenia, encephalitis, and gastrointestinal symptoms, which results in multiple-organ failure with a lethality rate ranging from 12 to 30% in the reported countries (Li et al., 2017).

Sun et al. (2014) showed that the recombinant envelope glycoprotein Gn of SFTSV binds to several kinds of SFTSV susceptible cells such as human umbilical vein endothelial cells and inhibits the infection of this virus to these cells. Importantly, glycoprotein Gn is confirmed to bind to NMHC IIA in immunoprecipitation coupled with the mass spectrometry assay (Sun et al., 2014). Supporting evidence for the involvement of NM IIA in SFTSV infection comes from a series of observations. The expression of NM IIA in susceptible cells incubated with SFTSV displays an initially increased and then decreased alteration when the cells pre-exposed to 4°C for 2 h are then shifted to 37°C for indicated time (i.e., 0–15 min) (Sun et al., 2014). It is suggested that SFTSV may utilize NM IIA for the entrance into the target cells, because 4 and 37°C are permissible temperatures for viral adsorption and penetration of the cell membrane, respectively. Notably, the total protein level of NM IIA is increased during viral infection (Sun et al., 2014). Additionally, application of different NM IIA inhibitors (including siRNA, anti-NM IIA antibody, and ML-7) can effectively suppress SFTSV infection (Sun et al., 2014). On the contrary, over-expression of NM IIA promotes the efficiency of SFTSV entry into HeLa cells that are less susceptible to this virus (Sun et al., 2014). These findings demonstrate that NMIIA is critical for the entry of SFTSV into target cells. However, it remains unidentified whether NM IIA plays other roles in the virus infection.

##### Porcine reproductive and respiratory syndrome virus (PRRSV)

Porcine reproductive and respiratory syndrome virus (PRRSV) is an enveloped and single-stranded positive sense RNA virus which belongs to the family *Arteriviridae*, within the order *Nidovirales* (Lunney et al., 2016). PRRSV is considered as one of the most significant pathogens that affects pigs globally, causing porcine reproductive and respiratory syndrome (PRRS) and leading to huge economic losses to the pork industry worldwide (Nieuwenhuis et al., 2012).

Consistent with the role that NM IIA plays as a receptor in HSV-1 infection. NM IIA has been identified to act as a receptor in PRRSV infection as well. Gao et al. (2016) found that the C-terminal domain of NM IIA interacts with glycoprotein GP5 of PRRSV at the early stage of its infection (binding and absorption). During the entry of PRRSV into the target cells, NM IIA is redistributed from the cytoplasm to the plasma membrane and its expression is increased. Suppression of NM IIA expression decreases the yield of PRRSV, while overexpression of NM IIA markedly contributes to viral infection. On the contrary, NM IIB has no these effects (Gao et al., 2016). Further research showed that PRRSV may make use of intercellular nanotubes for the transmission of virions from cell to cell, and the inhibition of NM IIA activity suppresses intercellular nanotube formation as well as the efficiency of intercellular spreading of virions (Guo R. et al., 2016). The latest research of Li et al. (2018) further confirmed that recombinant NM IIA C-terminal domain can block PRRSV infection via interaction with viral glycoprotein GP5. These observations suggest that NM IIA plays multiple roles in the infection of PRRSV at these critical steps such as the entry, cellular transport, even egress of this virus. Therefore, it is believed that NM IIA may directly contribute to the pathogenesis of PRRSV and be utilized as an important target for the development of potential agents against the viral infection.

##### Rice dwarf virus (RDV)

Rice dwarf virus (RDV), a non-enveloped and double-stranded RNA virus that belongs to *Phytoreovirus*, multiplies in plants as well as invertebrate insect vectors (Wei et al., 2008b). RDV was first discovered in Japan in 1883, and then widely spread in Asian countries including China, Korea, and Nepal, where it causes the loss of rice production. Pns 10 is a non-structural protein responsible for forming tubules which is involved in RDV intercellular spread (Wei et al., 2006). By using a selective myosin motor activity inhibitor (BDM), Wei et al. (2008b) found that the formation of Pns 10 tubules is prevented and the intercellular spread of RDV is inhibited. Similarly, the formation of RDV spherical structures is also inhibited (Wei et al., 2008a). These lines of evidence indicate that the myosin motor (NM II) does mediate RDV infection. However, when NM II executes its fundamental role, i.e., providing force for intracellular transportation, or whether NM II is involved in other processes of viral infection, remains unsolved.

### Bacteria-Triggered Discords

#### *Listeria monocytogene*s

*Listeria monocytogenes* (*L. monocytogenes*) is a Gram-positive, non-sporulating and rod-shaped bacterium belonging to the family *Acidobacteriaceae.* It is regarded as a food-borne pathogen with 13 serotypes based on their antigenic diversity. *L. monocytogenes* affects humans globally and causes low morbidity but high mortality (Radoshevich and Cossart, 2017). The clinical symptoms such as gastro-enteritis, bacterial sepsis and meningitis or abortion, vary with the physical conditions and ages of humans, and ingestion of the content of *L. monocytogenes* (Chen et al., 2017; Radoshevich and Cossart, 2017).

Plasma membrane blebs relying on NM II activity are the positive factor for pathogen dissemination (Fackler and Grosse, 2008). Furthermore, NM IIA might have multiple roles in the reaction of intracellular *L. monocytogenes* infection. For instance, Mesquita et al. (2017) found that listeriolysin O (LLO) induces the reorganization of the NM II A network into cortical bundles for the formation of plasma membrane blebs, whereas NM IIA protects plasma membrane integrity against LLO intoxication during *L*. *monocytogenes* infection (Mesquita et al., 2017). Almeida et al. (2015) also reported that NM IIA is tyrosine-phosphorylated by the Src tyrosine kinase in response to several bacterial pathogens. Importantly, the intracellular level of *L. monocytogenes* was found to positively correlate with the level of NM IIA activity or expression, because the inhibition of NM IIA activity by its inhibitor or the prevention of NM IIA tyrosine-phosphorylation or the depletion of NM IIA expression using siRNA, limited the infection of *L. monocytogenes*. Though in-depth study is required, these facts do confirm the involvement of NM IIA in the infection of these bacterial pathogens (Almeida et al., 2015).

#### *Neisseria gonorrhoea*e

*Neisseria gonorrhoeae* (*N. gonorrhoeae*) is a Gram-negative, non-flagellum and globular bacterium belonging to the family Neisseria bacteria. This pathogen is regarded as a crucial sexual concern because of its worldwide distribution, high susceptibility as well as widespread antimicrobial resistance (Berntsen et al., 2017). As a special human pathogen, *N. gonorrhoeae* infects the mucosal surface of humans via sexual transmission and causes gonorrhea (Wang et al., 2017).

*N. gonorrhoeae* infection alters actin reorganization, in which NM II plays multiple roles (Wang et al., 2017). As the first line of defense, the epithelial cells protect the host against the invasion of *N. gonorrhoeae*, whereas, *N. gonorrhoeae* infection induces exfoliation of endocervical epithelial cells. Wang et al. (2017) discovered that activated NM II is accumulated and redistributed to the *N. gonorrhoeae* adherent sites during their interaction. Inhibition of Ca^2+^ or myosin light chain kinase (MLCK)-dependent NM II activity decreases the percentage of epithelial exfoliation during *N. gonorrhoeae* infection. Additionally, the ability of *N. gonorrhoeae* to transmit across target cells, to penetrate into target cells and to induce the disassembly of junctions, is also suppressed (Wang et al., 2017).

## NM II Inhibitors With Potential Drug Application

Since most of the microbial infections discussed in this review lead to increased expression and/or activity of NM II, one would imagine that inhibiting its expression and or activity could result in decreased infection by microbials. It becomes clear that there are two ways for NM II inhibitors to affect the functions of NM II. The first one is to interfere with NM IIA expression at the post-transcriptional level, including microRNAs (e.g., let-5p-7f) and siRNA. The other one is to suppress NM II activity via the inhibition of MLCK or the ATPase, which is mediated by a series of inhibitors such as ML-7, ML-9, BDM, and Blebbistatin (see Table 2). Despite tremendous progress in identifying and developing NM II inhibitors in recent years, these NM II inhibitors have not been applied to drug development and clinical conditions. The unsolved problems mainly include: (a) the novel NM II inhibitors (microRNA and siRNA) show excellent specificity to the targeted MYH9 mRNA gene and down-regulate NM IIA expression. However, the potential toxicity (Guo J. et al., 2016) and off-target effects (Farooqi et al., 2018) limit their further application; (b) those traditional inhibitors, which are commercial and can be easily obtained, have low specificity, potency, and solubility, as well as (photo) toxicity, and furthermore interfere the activity of all three isoforms of NM II (Roman et al., 2018), restrict their further application.

**Table 2:** NM II inhibitors with potential drug application.

NM II inhibitors	NM II isoforms	Chemotherapeutic effects	Microbial-triggered discords (references)
MicroRNAs (let-7f-5p)	NM IIA	Directly bind to 3′ UTR of MYH9 gene	PRRSV (Li et al., 2016)
SiRNA	NM IIA NM IIB	Act on the target mRNA	SFTSV (Sun et al., 2014)
ML-7 and ML-9	NM II	Potently inhibits MLCK activity	HSV-1 (Arii et al., 2010)
BDM	NM II	Potently inhibits actin-activated ATPase activity	EHV-1 (Cymerys et al., 2016)
Blebbistatin	NM II	Potently inhibits ATPase activity	EHV-1 (Cymerys et al., 2016), HSV-1 (Antoine and Shukla, 2014), and KSHV (Valiya Veettil et al., 2010)


There are mainly two kinds of kinase phosphorylation that modulates NM II activity, namely MLCK and Rho-associated kinase (ROCK), both MLCK and ROCK act on the myosin light chain molecule at the residues of Thr18 and Ser 19 (Croft et al., 2005). In addition, the regulation of NM II ATPase can also affect its activity (Kim et al., 2008). Traditional NM II inhibitors, including ML-7, ML-9, BDM, and Blebbistatin, usually act via affecting MLCK, ROCK, or ATPase activity. Newly emerging NM II inhibitors (such as microRNA and siRNA) act via modulating the expression of NM II. Application of NM II inhibitors is a good strategy for determining the roles of NM II in cultured cells and tissues under the conditions of microbial infection. More importantly, these NM II inhibitors might be applied as potential drugs/vaccines in treating/preventing these diseases. NM II inhibitors, their mechanisms of action, and chemotherapeutic effects are summarized in Table 2.

### Novel Inhibitors

#### MicroRNAs

MicroRNAs (miRNAs) are a family of endogenous, non-coding small RNAs which typically consist of 22 ∼ 24 nucleotides, taking part in regulating gene expression by binding to corresponding mRNA to suppress its formation or translation. Growing evidence indicates the emerging roles for miRNAs in various pathological processes including viral infection and mycoplasm infection (Zhao et al., 2017; Zheng et al., 2018). For example, miRNA let-7f-5p is found to inhibit PRRSV infection via suppression of NM IIA expression which is mediated by directly binding to the 3′ UTR of its target MYH9 mRNA (Li et al., 2016). Therefore, overexpression of let-7f-5p could be a good way to suppresses PRRSV replication in infected cells (Li et al., 2016). However, whether this miRNA can be used as a target for the development of clinical drug/vaccine against PRSSV or the treatment/prevention of other NM II-related microbial infection, and the way to deliver miRNA into target tissues, requires further investigations.

#### Small Interfering RNA (SiRNA)

Small interfering RNA (siRNA), belonging to the family of RNA interference (RNAi), is a double-stranded RNA which typically consists of 20 ∼ 25 nucleotides. In recent years, siRNA has been widely employed as an effective tool for exploring gene function and drug targets. Based on its unique role in triggering the cleavage of target mRNA (Meng and Lu, 2017), it has been used to inhibit viral infection. For instance, the use of siRNAs targeting NM IIA decreases SFTSV infection efficiency by over 75%, and silencing NM IIB expression via siRNA reduces SFTSV infection efficiency by 11% (Sun et al., 2014). However, application of siRNA targeting NM IIA in RAW264.7 macrophage cells not only suppresses phagosome acidification and recruitment of LAMP-1, but also impairs the ability of host cells to defend against *Escherichia coli* infection (Gomez and Descoteaux, 2018). These facts suggest that the requirement for NM IIA in host defense against infections may depend on the types of microbials. It seems that downregulation of NM IIA alleviates viral infection but promotes the susceptibility to bacteria, respectively (Sun et al., 2014). Whether this is a general rule warrants further investigation.

### Traditional Inhibitors

#### ML-7 and ML-9

Both ML-7 and ML-9 are chemical products of naphthalene sulfonamide, which are effective NM II inhibitors via blocking MLCK (Shi et al., 2007; Arii et al., 2010). Compared with ML-9, ML-7 has been used more widely in experimental studies and clinical applications because of its higher inhibiting effect on smooth muscle MLCK and other MLCK isoforms (Saitoh et al., 1987; Xiong et al., 2017). Arii et al. (2010) found that ML-7 reduces Vero cell susceptibility to HSV-1 infection at the virus entry level by inhibiting NM IIA activity. Moreover, treatment of the murine model with ML-7 before viral inoculation could significantly inhibit HSV-1 infection and improve survival rate. Therefore, both ML-7 and ML-9 are used as novel therapies to block HSV-1 infection by inhibiting MLCK activity (Antoine and Shukla, 2014).

#### BDM

BDM is an actin-activated myosin ATPase inhibitor, which has been widely applied to cell biological studies (McKillop et al., 1994). As a small molecule, BDM has similar effects to ML-7 in some cases (Forer and Fabian, 2005). For example, both BDM and ML-7 could reduce the efficiency in the protein delivery from Golgi bodies to the endoplasmic reticulum in cells (Durán et al., 2003). When BDM was applied to cultured cells, Cymerys et al. (2016) revealed that progeny virions of EHV-1 could not “escape” from the infected cells, and transmission of virions from cell to cell was inhibited as well. Thus, it is suggested that BDM can be used as a drug for treating NM II-related viral infection.

#### Blebbistatin

Blebbistatin is a small molecule NM II inhibitor which shows high affinity and selectivity (Kovács et al., 2004). This inhibitor specially binds to the ATPase site of NM II heads and decreases the phosphate release rate, thereby suppressing the activity of NM II (Kovács et al., 2004). In recent years, Blebbistatin has been widely applied in many viral researches including EHV-1 (Cymerys et al., 2016), HSV-1 (Antoine and Shukla, 2014), HIV (Kadiu and Gendelman, 2011), and KSHV (Valiya Veettil et al., 2010). For example, Blebbistatin interferes with the KSHV and HSV-1 internalization process to inhibit the entry of virions (Valiya Veettil et al., 2010; Antoine and Shukla, 2014). Additionally, Blebbistatin could also inhibit EHV-1 intercellular spread (Cymerys et al., 2016).

## Summary

Microbial infection is a complex process, which needs to utilize a series of receptors and co-factors on/in the cells for microbial entry, then pathogens multiply inside and release their descendants outside the cell (Tomlin and Piccinini, 2018). In this review, we have comprehensively summarized the essential roles of NM II in diverse microbial-triggered discords, which occur in human beings (Valiya Veettil et al., 2010; Hays et al., 2012; Sun et al., 2014), animals (Cymerys et al., 2016; Gao et al., 2016), and plants (Wei et al., 2008b). Interestingly, most of these reported microbial infections can similarly result in NM II alterations, mainly leading to the up-regulation of NM II expression or activity (see Table 1). Developing NM II inhibitors would be a considerable therapy against microbial infection. Therefore, we also in this review discuss the recently reported NM II inhibitors which are applied to the investigation of the effects and the underlying mechanisms of NM II in microbial-triggered discords. These NM II inhibitors have been applied to demonstrating the effect on resisting microbial infections *in vitro*, even *in vivo* (Gao et al., 2016).

Notably, we are still at the beginning of understanding the roles of these important cellular skeleton proteins which are ubiquitously expressed in almost all cell types. Of these skeletal proteins, lines of evidence indicate that NM II acts as a crucial receptor/co-factor for microbial infection, which is mainly confirmed via two methods. The first one is to investigate the expression or activity of the members of the NM II family during microbial infection, the second is to analyze virus infection after the application of NM II inhibitors. However, understanding the precise mechanisms of NM II in microbial infection is ambitious, understanding how altered NM II activity or expression contributes to microbial infection may open a novel approach against these discords if the following questions are well solved: these include what the exact mechanisms of microbial infection leading to the abnormality of NM II activity or expression are; what the exact roles of NM II in the distinct processes of virus infection are; how we can improve the specificity of NM II inhibitors and reduce the side-effects of them and how many virus types there are in which NM II is involved in the infection. In spite of these unsolved problems, NM II does have the potential as a therapeutic target. For example, Blebbistatin (NM II activity inhibitor) was confirmed to inhibit PRRSV infection both *in vitro* (cell model) and *in vivo* (piglet model) without considering its side-effects (Gao et al., 2016). Indeed, due to its properties such as poorly water soluble, cytotoxic, and prone to (photo) degradation, the wide applicability of Blebbistatin is hindered (Rauscher et al., 2018). These facts suggest Blebbistatin is still druggable if enough efforts are taken to improve these adverse features. Meanwhile, *in vitro* studies also demonstrated that downregulated expression of NM II using miRNA or siRNA approach effectively prevents the infection of PRRSV and SFTSV (Sun et al., 2014; Li et al., 2016), whereas *in vivo* evidence conducted in knockdown or knockout animals is still lacking. Therefore, further investigations are required to provide substantial facts.

In summary, NM II, as a molecular motor, plays a major role in cell movement as well as other physiological activities. Confronted with many microbial infections, collective evidence clearly confirms that NM II may act as an important receptor/co-factor and play multiple roles, such as microbial entry, replication and release (Cymerys et al., 2016; Gao et al., 2016). However, our knowledge of the precise mechanisms remains obscure. More detailed understanding of the interaction between NM II and microbial infection should be further explored in this field, which would provide new insights for developing special NM II inhibitors as promising drugs for the control and treatment of these and other microbial-triggered discords at a clinical level, which differs from the traditional therapeutic strategy.

## Author Contributions

LT contributed to the development and writing of the paper, reviewing relevant literature, and preparation of tables in the paper. XY and XC contributed to the writing of the paper and provided suggestions on the revision. YL contributed to the drawing of the figure. SG and AW provided substantial, direct, and intellectual contribution to the work. All authors approved the article for publication.

## Conflict of Interest Statement

The authors declare that the research was conducted in the absence of any commercial or financial relationships that could be construed as a potential conflict of interest.
